# The impact of the competence quorum sensing system on *Streptococcus pneumoniae *biofilms varies depending on the experimental model

**DOI:** 10.1186/1471-2180-11-75

**Published:** 2011-04-14

**Authors:** Claudia Trappetti, Luciana Gualdi, Lorenzo Di Meola, Prashant Jain, Cindy C Korir, Paul Edmonds, Francesco Iannelli, Susanna Ricci, Gianni Pozzi, Marco R Oggioni

**Affiliations:** 1 Dipartimento di Biotecnologie, LAMMB, Policlinico Le Scotte (lotto 5 piano 1), Universita di Siena, 53100 Siena, Italy; 2 School of Biology, Georgia Institute of Technology, 310 Ferst Drive, Atlanta, Georgia 30332, USA; 3 Research Centre for Infectious Diseases, School of Molecular and Biomedical Science, University of Adelaide, South Australia, 5005, Australia

## Abstract

**Background:**

Different models for biofilm in *Streptococcus pneumoniae *have been described in literature. To permit comparison of experimental data, we characterised the impact of the pneumococcal quorum-sensing competence system on biofilm formation in three models. For this scope, we used two microtiter and one continuous culture biofilm system.

**Results:**

In both microtiter models the competence system influences stability and structure of biofilm in the late attachment phase and synthetic competence stimulating peptide (CSP) restored wild type phenotypes in the *comC *mutants unable to produce the peptide. Early attachment of single cells to well bottoms was found for both systems to be competence independent, while later phases, including microcolony formation correlated to an intact competence system. The continuous culture biofilm model was not affected by mutations in the competence locus, but deletion of capsule had a significant impact in this model.

**Conclusions:**

Since biofilm remains a largely uncharacterised multi-parameter phenotype it appears to be advisable to exploit more than one model in order to draw conclusion of possible relevance of specific genotypes on pneumococcal physiology.

## Background

*Streptococcus pneumoniae *is one of the main bacterial pathogens causing acute invasive disease, including meningitis, sepsis and community acquired pneumonia. Pneumonia, of which the pneumococcus is the leading cause, still accounts worldwide for over 150 million clinical episodes yearly, which contribute to approximately 1.9 million deaths [1]. Even more frequent are non-invasive pneumococcal acute conjunctivitis and otitis media. Pneumococci are also part of the normal flora of humans, as they colonise the nasopharynx soon after birth and carriage is reported to be self limited to periods from few days to few months [2,3]. Successive carriage episodes are generally due to strains of different capsular types. Progression to invasive disease occurs within the first weeks of carriage [2]. Recently, interest has been raised on physiology of bacteria in different niches of their natural environment: the human host. Direct microscopy analysis, carried out on human biopsy specimens of the sinus and the middle ear mucosa and the adenoids showed the presence of pneumococcal cells embedded in extracellular matrix indicative of microbial biofilms [4-6]. Recently, the presence of biofilm-like structures in the lungs of animals infected with *S. pneumoniae *was also documented [7]. These studies provided important evidence that pneumococci in different diseases are not behaving as planktonic cells, but predominantly show characteristics of a biofilm like state.

Pneumococcal animal models of disease as well as models of carriage have been associated to biofilm-like infections [8-13]. It has been shown that gene expression of pneumococci during infection of lungs and meninges in mice was comparable to that of pneumococcal biofilms [8]. In this model the development of biofilm depended on the competence system, and the addition of the competence stimulating peptide (CSP) to the medium was necessary for biofilm formation. The direct association of the competence system to pneumococcal disease was demonstrated by the fact that virulence in sepsis and pneumonia could be modulated by CSP and by showing increase of disease severity in mice directly challenged with biofilm cells [8,14]. The correlation of biofilm to carriage was confirmed by mutants that produced less biofilm in an *in vitro *model and also showed reduction in their colonisation capacity [9]. Recent data from our group showed that free sialic acid in culture medium represents the signal necessary for biofilm formation. Furthermore, this signal increases pneumococcal colonisation and translocation to the lung in mouse models of carriage [10]. It is of interest to underline that despite existence of pneumococcal biofilms in humans and correlation between virulence in experimental infection models and aspects of biofilm, so far no important correlation of pneumococcal clinical isolates, clones, serotypes, or MLST types to their capacity to form *in vitro *a biofilm was shown [15,16].

Biofilm models are less standardised than the classical mid log growth phase, in which most microbiological research has been done. Thus there is an urgent need to identify the most appropriate biofilm model systems in order to obtain significant and comparable results. In pneumococci the description of a continuous culture model provided for the first time a simple approach for studying biofilms [17]. This work followed earlier descriptions of biofilms grown on sorbarod filters [18,19]. The continuous culture model demonstrated growth of pneumococci up to seven days and the production of an extra cellular matrix polysaccharide [17]. This work stimulated active research in the field of pneumococcal biofilm. Work included more extensive descriptions of the architecture, and the changes that occur upon continuous culture biofilm development [20], as also characterisation of phenotypes of colonies grown from cells detached from a biofilm [21,22]. Finally simplified models for static bacterial biofilms in microtiter plates were also set up [8,10,13,23,24]. Formation of pneumococcal biofilm formation was since then reported by many researches [7,15,16,25-27]. So far the two regulatory systems demonstrated to influence biofilm formation in pneumococci, both *in vitro *and *in vivo*, were the competence regulatory system and sialic acid metabolism [8,10,28]. Still, as in all other work on pneumococcal biofilm, only a single *in vitro *model were used for description a given phenotype or event. In the present work we perform a more detailed analysis of the influence of competence on pneumococcal biofilm and extend the assays to three different biofilm models. These studies are aimed to provide tools and knowledge that may facilitate comparison of literature data and help selection of the most suitable systems for pneumococcal biofilm research.

## Results

### Microtiter biofilm model with exponential growth

**Figure 1:**
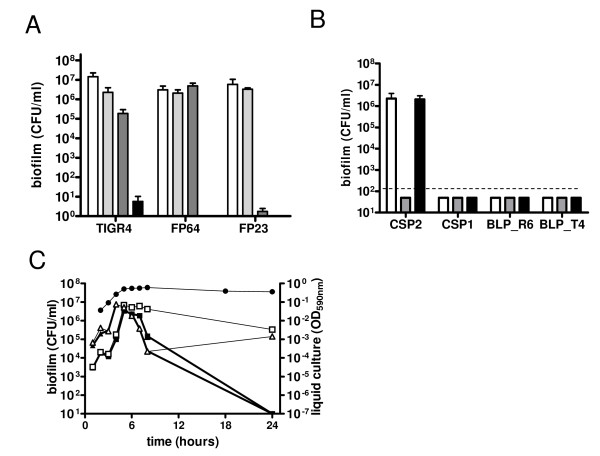
**Characteristics of the biofilm model based on exponentially growing cells**. Pneumococcal attachment to 96-well microtiter wells after 1:100 dilution in TSB medium was evaluated by viable counts following detachment of cells by sonication. Prior to sonication 18 hour biofilm was washed three times with medium. Panel A reports the effect of the duration of sonication (2 sec. white; 5 sec. light grey; 10 sec dark grey; 30 sec. black) on detachment and survival of pneumococcal cells. Panel B reports biofilm formation of TIGR4 (open bar), FP184 (mutated for *comD *response regulator; grey bar) and FP218 (mutant of response regulator of the BLP system; black bar) in media supplemented with CSP2, its allelic variant CSP1, BLPTIGR4 or its allelic variant BLPR6. Panel C shows a time course experiment with simultaneous evaluation of turbidity of the planktonic culture (closed circle; OD values of TIGR plotted on right axis) and biofilm counts using encapsulated TIGR4 (square) and its rough isogenic mutant FP23 (triangle). Experiments were performed in TSB supplemented with CSP2 (open symbols) or plain TSB (closed symbols). Turbidity data are form strain TIGR4. Data are from quadruplicate experiments (the small SD are not visible due to log scale of the graph)

We have previously described the importance of competence system in a model of pneumococcal biofilm based on low numbers of cells inoculated in undiluted growth medium [8]. In this model, a 1:100 inoculum of frozen mid-log cells enabled exponential growth of pneumococci in the microwell. To monitor the cells attached to surfaces we performed viable cell counts after detachment from the plastic by sonication. Pneumococcal cells were efficiently recovered after only 2 sec of sonication. Control of the method showed that the two encapsulated strains showed a higher resistance to killing by ultrasounds than the rough mutant (Figure 1A). Microscopic examination revealed complete detachment of cells without evidence of clumping of detached cells, indicating that CFU enumeration is a suitable approach for cell count (data not shown).

Pneumococcal biofilm formation on microtiter plates was described to be dependent on the addition of CSP to the growth medium [8]. In the present work we analyze the dynamics of pneumococcal biofilm formation on flat bottom polystyrene wells. To describe the formation of biofilm over time we harvested pneumococci at different time points and compared the viable counts of bacteria in the medium to those of cells detached from the surface of the microtiter wells. During the first hours of the experiment attachment increased approximately proportional to the increase in cell density of planktonic cells (Figure 1C). In correspondence of late exponential growth (after 4 h of incubation) the number of attached cells rose by hundred to thousand fold within on-two generations and then the number of attached cells remained stable for 2 - 3 h (corresponding to early stationary phase). After this period a decrease in the number of attached viable cells was evidenced and only in the presence of CSP attached pneumococci could be recovered after 24 hours. Data show that during this first 8 h of incubation the presence of CSP did not influence pneumococcal attachment, whereas CSP was crucial for cell attachment at later time points. Performing this assay with wild type (*wt*) and un-encapsulated mutants in parallel, gave identical results (Figure 1C). Control experiments carried out by adding CSP after the first 8 hours of incubation yielded no detectable biofilm counts at 24 hours for both TIGR4 and FP23 (only 1 CFU in a total of 4 microtiter wells for TIGR4; no CFU recovered for FP23), which equals to the data without any addition of CSP (Figure 1C).

**Figure 2:**
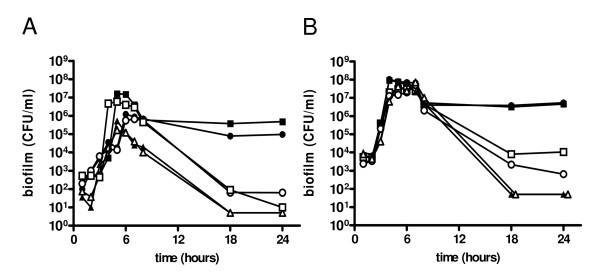
**Dynamics of biofilm formation in the model based on exponentially growing cells**. Biofilm formation in *comC *and *comD *mutants in different genetic backgrounds. Biofilm formation in microtiter plates was evaluated in the presence (closed symbols) and absence of CSP (open symbols). Rough *wt *pneumococci (squares), the mutants for *comC *encoding CSP (circles) and for *comD *encoding the CSP-receptor histidine kinase (triangles) were assayed in parallel in a time course experiment. Panel A: Biofilm formation induced by CSP2 in strains derived from strain TIGR4 (*comC*2, *comD*2). Mutants assayed were FP23 (non-capsulated TIGR4) and its derivatives FP64 (*comC *mutant) and FP184 (*comD *mutant). Panel B: Biofilm formation induced by CSP1 in strains derived from D39 (*comC*1, *comD*1). Mutants assayed were RX1 (non-capsulated mutant) and its derivatives FP5 (*comC *mutant) and FP48 (*comD *mutant). Data of the twelve time course experiments are from one representative series; repetition showed comparable results.

To better characterize a competence depended-biofilm, we performed a similar experiment using a *comC *deletion mutant (FP64), unable to synthesize CSP but still responsive to exogenous CSP, and a *comD *mutant (FP184) unable to sense CSP [29]. As shown in Figure 2A both strains did attach to the plastic during the first hours of incubation independently from the addition of CSP, confirming that the first phase of biofilm formation is CSP independent. In contrast maintenance of biofilm for prolonged incubation times, for both the *wt *and *comC *mutant FP64, was completely dependent on addition of synthetic CSP. In contrast the CSP receptor *comD *mutant (FP184) could not be complemented by addition of synthetic peptide [8,14]. Microscopic examination at 18 to 24 hours showed absence of any biofilm-like structure in this condition. To confirm that the phenomena observed was serotype independent, we performed the same experiment using the RX1 strain, a D39 derivative carrying the *comCD*1 allele and responsive to CSP1 (Figure 2b). As in TIGR4, there were two distinct phases of biofilm formation and maintenance, respectively independent and dependent from competence. As described above also the D39 *comD *mutant resulted impaired in biofilm maintenance even in presence of CSP. Repetition of experiments with an unrelated *comD *deletion mutant in (FP421) yielded at 24 hours no detectable biofilm counts, as for the insertion mutant. These data confirm that the first phase of biofilm formation is competence-independent, while the second phase is competence-dependent.

To test the specificity of CSP effect on biofilm formation of the TIGR4 strain, carrying the *comCD*2 alleles, biofilm formation was assayed with CSP1 and CSP2 [30]. Incubation with CSP2 yielded biofilm counts of 10^5 ^CFU/well after 18 hours of incubation (Figure 1B). No cells were recovered when incubating without CSP or with CSP1 (Figure 1B). In parallel to TIGR4, biofilm formation was also assayed with FP218, a mutant for the response regulator of the related Blp bacteriocin peptide sensing system [31-33]. Incubation of FP218 with CSP2 yielded biofilm counts of 8 × 10^4 ^CFU/well, while no biofilm was detected after incubation with CSP1, the BlpC peptide of TIGR4 or the BlpC peptide of R6 (Figure 1B).

### Stationary phase type microtiter biofilm model

**Figure 3:**
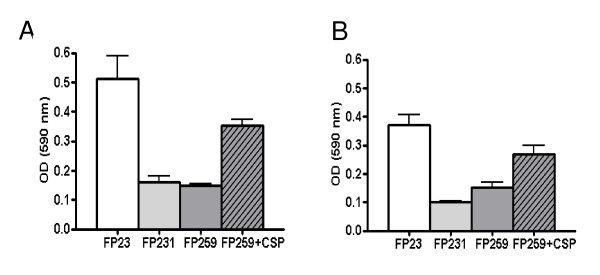
**Impact of competence in the stationary phase type microtiter biofilm model**. In this model, biofilm formation was evaluated by both crystal violet staining and analysis at the spectrophotometer. The FP23 strain (non-capsulated TIGR4) was compared with its isogenic mutants in *comD *(FP231) and *comC *(FP259). The *comC *mutant FP259 was also assayed with addition of synthetic CSP to the medium (striped bars). The experiment was performed in BHI and read after 24 (panel A) or 48 hours (panel B) of incubation at 37°C. The differences in biofilm formations between the *wt *and the *comC *and *comD *mutants and between FP259 with and without CSP were statistically significant (p < 0.005). Data are from triplicate experiments.

Many biofilm models already described in the literature rely on high inocula of cells into poor media [7,15,23-25]. To compare our data reported above, we set up this model for pneumococcal biofilm. Pneumococcal cells grown to early stationary phase were harvested, washed and inoculated 1:10 to approximately 5 × 10^7 ^CFU/ml into diluted or undiluted medium in microtiter wells [24]. To permit extension of the experiment for several days half of the spent medium was exchanged twice daily with fresh medium. In this setup the utilisation of diluted fresh medium did not reduce significantly pneumococcal attachment (data not shown) and variation of medium form TSB to BHI yielded approximately the same results (data not shown). Due to the high inoculum cells didn't go through exponential phase of growth, but maintained constant cell density in the liquid phase (data not shown). In this series of experiments the biofilm formation was quantified through spectrophotometic analysis of crystal violet stained biofilm cells. This readout was chosen since pneumococci tended to form aggregates on the well bottom (see below) and sonication at sub-lethal doses was not sufficient to ensure their disggregation, rendering viable counts a non reliable parameter (data not shown). A biofilm formed in such conditions could be maintained for up to 5 days, with little changes due to dilution of the medium (data not sown), in accordance with what has been reported by others [24]. To test the impact of competence in this model we analysed the same series of *wt *and *comD *and *comC *mutants as above. As shown in Figure 3A, the *wt *strain produced significantly more biofilm than the two competence mutants at 24 h. Supplementation of the medium with synthetic CSP complemented the phenotype of reduced biofilm formation in the *comC *mutant. When analysing the biofilm formation after 48 hours of incubation, we observed an identical trend (Figure 3b).

**Figure 4:**
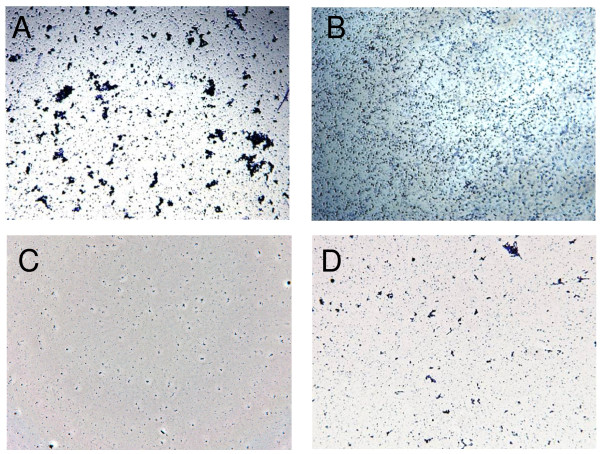
**Microscopy of cells in the stationary phase microtiter biofilm model**. The images (40 × magnification) show the attachment of pneumococci to the surface of microtiter plates after 24 hours of incubation. The *wt *strain TIGR4 (panel A), the *comD *mutant (panel B), the *comC *mutant (panel C) and the *comC *mutant with the addition of CSP2 (panel D) were compared. Biofilm images are taken on crystal violet stained cells observed in bright filed using the 40 × objective of a Leica DM1000 Microscope and a DFC digital camera.

To explain these differences microscopy was performed. The images reported in Figure 4A show biofilm formed by the TIGR4 strain and the *comC *and *comD *mutants (Figure 4B and 4C). The addition of CSP to the *comC *mutant increase the number of cells attached (Figure 4D). More striking was the observation that *wt *cells formed microcolony-like aggregates on the well bottom, which increased in size and number over time (data not shown). Interestingly these microcolonies or aggregates were lacking in the *comD *and *comC *mutants, but could be again observed in the CSP complemented *comC *mutant (Figures 4B-D). None of the assayed strains examined did aggregate in suspension. D39 and its derivatives showed similar structures as observed in the TIGR4 background (data not shown).

### Continuous culture biofilm

We used the continuous flow biofilm model system developed by CDC [17] to evaluate growth and biofilm formation of three *S. pneumoniae *strains (TIGR4, FP184, and FP23). The current study was performed with a bioreactor containing eight removal rods, each of which held three removable coupons. After inoculation, the reactor was operated in batch mode for 12 hours, after which continuous flow was initiated. Planktonic and biofilm samples were collected at 12 hour intervals for 48 hours, respectively form the outlet drainage tubing and by scraping the surface of the coupons [17]. Direct samples were utilised for CFU enumeration, formalin fixed samples for microscopy and frozen samples for RT PCR.

**Figure 5:**
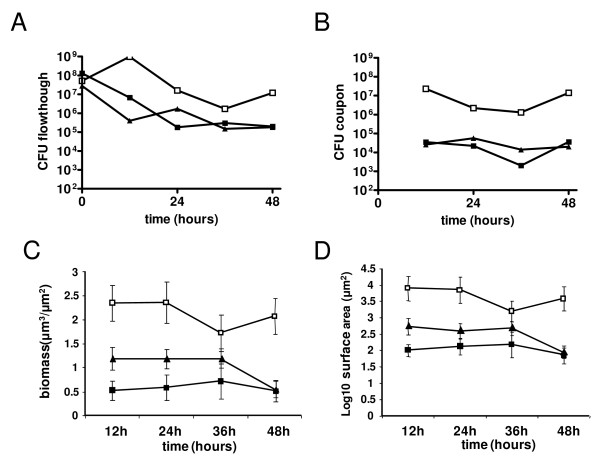
**Biofilm formation on coupons in the continuous culture biofilm model**. Continuous culture biofilm was analysed for TIGR4 (closed square), its rough mutant FP23 (open square) and the *comD *mutant FP184 (closed triangle). Bacterial counts in flow through (panel A) and on the coupon (panel B) are from a single experiment while data on biomass (panel C) and the surface area of the biofilm (panle D) are from 15 measurements at each timepoint. Biofilm samples grown on polycarbonate disks were collected at 12, 24, 36, and 48 hours and fixed in formaldehyde. Biofilm was stained with Sybr Green I, a general double stranded DNA stain, and examined with a Zeiss epifluorescence microscope with an ApoTome attachment. Image stacks through the depth of the biofilm were obtained (Z-stacks) from each disk, with 5 stacks per disk and 3 disks per time point and per strain. Biomass in each image stack was enumerated in the COMSTAT image analysis program. Data was transformed by multiplying each point by 10,000 and obtaining the log (base 10) value.

In continuous culture biofilm the quantity of cells in the flow through and attached to the coupons was stable over time with biofilm counts being generally 10 to 100 fold lower than planktonic cells (Figure 5A-B). Data from analysis of biofilm cell counts, thickness and surface area concorded and showed higher values for the rough FP23 strain than for the *wt *TIGR4 strain and it's isogenic *comD *mutant, which in turn did not differ significantly (Figure 5A-D). These data clearly show an absence of a competence related phenotype in this model while suggesting that for this model capsular polysaccharide has a significant impact on bacterial adhesion to the coupon.

### Gene expression analysis

In order to compare the level of expression of competence genes in the biofilm models we analysed the pattern of relative gene expression by real time PCR [8,10]. All data are reported as fold change in gene expression with respect to exponential planktonic cells. The expression of the competence genes *comA*, *comE *and *comX *showed respectively 15 (p < 0.05), 25 (p < 0.01) and 23 (p < 0.01) fold increase in the biofilm model with exponential growth, 23 fold (<0,05) and 49 fold (<0,001) in the Stationary phase type microtiter biofilm model (no data on *comE*) and 7.6 (non significant), 20 (p < 0.05) and 16 (p < 0.001) fold increase in the continuous culture model. Quantification of the capsule operon expression monitoring *cpsA4 *showed no variation in any model, while expression of the neuraminidase regulon, monitored on *nanA *and *nanB *was significantly upreguleted in biofilm (data not shown). Among the genes assayed, pneumolysin showed higher expression in planktonic cells compared to biofilms in both models, and the capsule showed no relative change in gene expression. The flow through of the biofilm reactor showed essentially the same expression profile as the control samples of exponentially growing cells.

## Discussion

Various biofilm models have been developed for *S. pneumoniae *over the last years including sorbarod filter models [18,19] and continuous culture reactor biofilms [17,20-22]. Simpler models rely on biofilms formed on microtiter plates, with or without exchange of culture medium [7-10,15,16,23,24,27,34]. Since no comparative analysis has previously been done, in this work we compare the impact of quorum sensing in three models.

We have previously described the importance of CSP addition to culture media to obtain stable biofilm after o.n. incubation using a narrow range of CSP concentrations in a model based on low multiplicity seeding of cells [8,34]. Here we show that pneumococci attach to surfaces during late exponential phase, and that this attachment is competence independent, while the stability of the sessile cell-community is dependent on the addition of exogenous CSP and a functional competence regulatory system. These results are in accordance with previous data on attachment to plastic surfaces influenced by sialic acid [10] and competence dependent late biofilm [8,34]. Attachment during late exponential phase of growth is in accordance with many models that identify the signal for formation of sessile communities in nutrient limitation or other stresses [10,27,35]. The fact that late addition of CSP has no effect on biofilm stability indicates that the competence-dependent events most probably occur prior, during or early after attachment.

When assaying for competence related phenotypes in the two other biofilm models, the effects of quorum sensing were different. The second microtiter biofilm model, more frequently used in pneumococcal research, relies on incubation of high numbers of stationary-phase cells [24]. In this model, the addition of synthetic CSP was not a necessary, however strains unable to synthesize or sense CSP were found to attach to a lower extent the surface compared to the *wt*. By microscopic analysis we verified that this phenotype was not due to a reduction in the number of single attached cells, but it was due to a reduction in number and size of surface attached microbial aggregates. Microcolony formation, already described as an important phenotype in pneumococcal biofilm [7,15,24], could be restored in *comC *mutant strains by addition of synthetic CSP to levels similar to *wt *strains.

The fact that none of the well known genes directly or indirectly regulated by competence has a direct link to attachment of biofilm underlines that effects seen in planktonic exponentially growing competent cells differ from the biofilm stabilisation phenotype seen here [36]. There are parallelisms between our findings and recent work in *S. mutans *where biofilm formation was also linked to the ComCDE system [37], although if genomic and genetic data indicate that the *S. mutans *ComDE is orthologous to the *S. pneumoniae *BlpRH system and does not directly control transformation [33,38]. Competence quorum sensing defects in *S. mutans *were found to determine reduction in biofilm biomass, and addition of CSP partially restored *wt *biofilm architecture [39]. In contrast to *S. pneumoniae *these ComCD-dependent phenotypes were correlated to the initial stages of biofilm development [39].

Biofilm microcolonies are examples of non-homogeneous microbial populations. In this context, our data indicate a significant effect of the competence quorum sensing system on the capacity of pneumococci to form these aggregates. Such aggregation behaviour in a non-homogeneous population is consistent with the observed clumping in a mixture of competent and non-competent cells which depends on the release of DNA into the medium [40,41]. Correlation of competence, cell clumping and DNA release fit well with the presence of DNA in the extracellular matrix of attached pneumococci and to subsequent sensitivity of pneumococcal biofilm to DNAse [23,24]. The release of DNA into the extracellular matrix through the endogenous CSP pathway has also been described to have a significant impact on biofilm biomass in *S. mutans *[42]. We lack a precise molecular characterisation of the events and we cannot exclude that some of the effects may be indirect and determined through an unknown regulatory pathway. The intriguing correlation of the pneumococcal serine-rich repeat protein (PsrP) to adhesion, aggregation and biofilm formation seems not to appear to show any obvious overlap to the observations reported in this work, inasmuch strain D39 and its derivatives do not carry the *psrP *operon [7]. The neuraminidase upregulation found in this work is also in accordance with the observed impact of sialic acid and the *nanAB *regulon on pneumococcal biofilm, even if again no obvious correlation can be drawn between the two putatively involved regulatory events [10]. In both cases, conditioning experiments may provide a useful approach to correlate phenotypes as shown in the related species *S. mutans *and the sialidase-positive *S. intermedius *[43,44].

In contrast to the two previous models, the continuous culture biofilm model gave a different result. Here the biofilm formation is not influenced by the competence system, despite gene expression analysis of the competence genes appears to be approximately the same in all models. In contrast to the microtiter models, the reactor model demonstrates a significant impact of the capsule. Decreased attachment of encapsulated strains is in agreement with data of others which carefully documented enhanced adhesion to surfaces and biofilm formation in rough strains [19,22,23,25,45].

## Conclusions

In conclusion our results demonstrate a significant effect of the pneumococcal competence system on biofilm in two out of three models highlighting the importance of the choice of the experimental model. It should also be noted that biofilm work, especially in a species like pneumococci undergoing stationary phase autolysis, relies on a methodology for which most parameters are unknown (generation time, homogeneity of the population, metabolism etc.) and where the results can be severly influenced by minor technical changes [46]. This should be taken into account, not only when assaying single mutants, but especially when running comparative assays on clinical isolates or mutant libraries [9,15,16]. Data here do not indicate superiority of any of the three models,. Each model has advantages and drawbacks, suggesting the use of different approaches in order to decipher different aspects of pneumococcal physiology.

## Methods

### Strains and growth conditions

Pneumococcal strains used in this work are reported in Table 1. Cells were grown in tryptic soy broth (TSB; Becton Dickinson), Brain Heart Infusion (BHI; Becton Dickinson) or tryptic soy agar (TSA) supplemented with 3% horse blood at 37°C in a CO_2_-enriched atmosphere. Bacterial stocks were prepared from mid log cultures and stored frozen at -80°C in 10% glycerol. When appropriate antibiotics were used at the following concentrations: kanamycin 500 μg ml^-1^, spectinomycin 100 μg ml^-1^, chloramphenicol 3 μg ml^-1 ^and novobiocin 10 μg ml^-1^.

**Table 1:** *S. pneumoniae *strains

Strain	Relevant properties	Mutated ORFs*	Reference
TIGR4	Type 4 strain, *comC2*-*comD2*	-	[52]
D39	Type 2 strain, *comC1*-*comD1*	-	[29,47,53]
Rx1	D39 natural mutant; Δ*csp2A-H*	spd0315-spd0323	[47,54]
FP5	Rx1 Δ*comC1*; Cm^R^	spr2043	[29]
FP23	TIGR4 Δ*cps4A-J*; Km^R^	SP0346-SP0366	[47,55]
FP48	Rx1 *comD1::aphIII*; Km^R^	spr2042	[8]
FP64	TIGR4 Δ*comC2*; Cm^R^	SP2237	[8]
FP175	TIGR4 *luxS::cat*; Cm^R^	SP0340	[56]
FP184	TIGR4 *comD2::aphIII*; Km^R^	SP2236	[14]
FP218	TIGR4 *blpH::ermB*; Em^R^	SP0527	this work
FP231	FP23 *comD2::aphIII*; Spe^R^	SP2184	this study
FP259	FP23 Δ*comC2*; Cm^R^	SP2185	this study
FP421	RX1 Δ*comD1*; Spe^R^	spr2042	this study

* numbering refers to the TIGR4, D39 and R6 genome sequences respectively

### Mutant construction

Isogenic mutants were constructed by gene SOEing as already described [47,48]. The TIGR4 capsule mutant FP23 has a deletion of the whole capsule locus, while the rough D39 derivative RX1 is a historical lab strain [47]. The *comC *mutants have all the identical in frame deletion of the *comC *gene, which is substituted by a chloramphenicol resistance marker [29]. The mutants differ inasmuch the cassettes for the two allelic variants of *comCD *were constructed separately [29]. The mutants for the CSP receptor histidine kinase carry both the same Mariner-transposon insertion within *comD *at nucleotide 152 [14]. An independent RX1 mutant was constructed by deleting most of the *comD *gene (*comD*_613-1168_:: *aad9*) to confirm phenotypes of the insertion mutant described above. The *blpH *deletion was amplified by PCR from mutant 486 hk (type 3 strain 0100993) [49] using primer 139 (TCCTTTAATCTGGGTGCCAGTCTT) and 140 b (GATATTGAACTGGGTATCACAAAGAC) and transformed directly into TIGR4 to yield FP218.

### Quorum sensing peptides

Peptides for assay of cell-cell signalling phenomena were obtained by Inbios (Pozzuoli, Napoli, Italy) as normal unmodified linear peptides of 95% purity. Peptides were CSP1 (EMRLSKFFRDFILQRKK), CSP_2 _(EMRISRIILDFLFLRKK), BlpC_TIGR4 _(GLWEDLLYNINRYAHYIT) and BlpC_R6 _(GWWEELLHETILSKFKITKALELPIQL). CSP1 and BlpC_R6 _are encoded respectively by *comC1 *and *blpC *of D39 (R6 genome) while CSP2 and BlpC_TIGR4 _correspond to the mature gene products of *comC2 *and *blpC *of TIGR4.

### Microtiter biofilm methodology: model based on diluted mid log phase inoculum

Cells were grown in 96-well flat-bottom polystyrene plates (Sarstedt, USA). For inocula frozen mid-log pneumococcal cultures were diluted 1:100 in 200 μl of TSB with addition of CSP. CSP1 was used at 30 ng/ml for D39 and its derivatives, while CSP2 at 100 ng/ml for TIGR4 and its derivatives. Plates were incubated at 37°C in a CO_2_-enriched atmosphere. Turbidity of bacterial cultures (OD_590_) was measured by using the VERSAmax Microplate Reader (Molecular Devices, Sunnyvale, Ca). To remove planktonic cells, wells were washed four times with ice-cold TSB, and added with 100 μl of TSB containing 10% glycerol. To detach biofilm cells, plates were sealed and floated on a sonicator water bath (Transonic 460, 35 kHz, Elma, Germany). Sonication times of 2, 5, 10, and 30 seconds were evaluated in preliminary experiments, while all following experiments were performed using 2 sec. Detached cells recovered from plates for CFU counts and quantitative real time RT PCR were stored frozen until use. Microscopic examination of attached pneumococci was done in 6-well plates added with 1 ml of medium and incubation in anaerobiosis. The use of 6-well plates allowed microscopic examination of cells at the bottom of wells using a normal light microscope (not inverted), since they permit insertion of the microscope objective within the wells.

### Microtiter biofilm methodology: model based on enriched stationary phase inoculum

Cells grown to early stationary phase were inoculated 1:10 into TSB or BHI medium, either undiluted, diluted 1:2, 1:3 or 1:4, with or without supplementation with CSP (concentrations as above) [24]. In this model biofilms were grown in 96 well plates for quantification only or in 6-well plates for microscopic examination. Plates were incubated at 37°C in a CO_2_-enriched atmosphere. To permit duration for more than 24 hours 50% of spent medium was exchanged twice daily with fresh prewarmed medium. AT the termination of the experiment wells were washed three times, and the biofilm was detected by crystal violet staining. Staining was done after desiccation at 50°C and staining with 1% crystal violet for 30 min followed by microscopic examination. For quantification stain was detached with 70% ethanol solution for 30 min and quantitative analysis was performed after transfer of the ethanol to a new mictotiter plate by measuring crystal violet absorbance at 590 nm.

### Continuous flow biofilm model

The continuous flow biofilm model system used in this work had been developed by CDC [17]. In the original work [17], the CDC bioreactor was connected to a FTIR laser spectrometer holding an attenuated total reflectance (ATR) flow cell. The current study was performed with the CDC bioreactor system alone. The bioreactor contained eight removal rods, each of which holds three removable polycarbonate coupons. Each coupon has a diameter of 1.3 cm which provides the surface for biofilm growth. Following assembly of the bioreactor, 400 ml of BHI broth supplemented with casein [0.5%] and yeast extract [0.2%] was added to the bioreactor and sterilized in an autoclave. Then, the bioreactor was placed in a Class II bioSafety Hood, and inoculated with 9 ml of a monoculture of the designated *S. pneumoniae *strain. Immediately, the inoculated bioreactor was placed in a water bath heater that maintained a temperature of approximately 35°C, and connected to a pre-sterilized carboy that contained 4 litre of 10% BHI plus supplements. During each experiment, the environment of the bioreactor was purged continuously with a filter-sterilized compressed gas mixture (5% oxygen, 10% Carbon dioxide, 85% nitrogen).

Immediately after inoculation, the reactor was operated in batch mode (closed system) for 12 hours, during which growth was agitated by a magnetic stirrer (Barnstead, Inc., Dubuque, IA) at 60 rpm. Continuous flow (open system) was initiated by pumping 10% BHI broth with a Masterflex peristaltic pump (Cole Parmer, Niles, Ill) at a flow rate of 0.5 ml/min to the bioreactor. Both planktonic and biofilm samples were collected at designated time periods. Three samples were collected at 12 hour intervals, and the duration of the experiment was 48 hours. (i) A planktonic sample (10 ml) was collected into a sterile test tube from an in-line switch of the outlet drainage tubing that connected the bioreactor to the waste carboy. (ii) Biofilm-associated cells were obtained by removing a single rod (containing two coupons) from the bioreactor. Then, biofilm-associated cells were collected by scraping the surface of each coupon separately into the same test tube with a sterile wood applicator, and rinsing intermittently with 9 ml of sterile Butterfield Buffer, and processed further by methods previously described [17]. Subsequently, viable cell counts (CFU/ml) were determined from the planktonic cell sample and from the biofilm-associated cell sample using the tube-dilution spread plate method. (iii) An additional rod (containing three coupons) was removed from the bioreactor at each sampling time period. Then, each coupon was removed, and placed directly in a designated well of a 12-well tissue culture tray, fixed with formalin, and stored at 4°C. Following the completion of each experiment, all fixed coupons were transported to the Centres for Disease Control for subsequent imaging of biofilm structures. Frozen samples were sent to Siena for RT PCR and matrix detection.

### RNA extraction, retrotranscription and quantitative real time RT-PCR

Sample preparation and real time RT PCR was essentially as already described [8]. RNA was extracted by using "SV Total RNA Isolation System Kit" (Promega) and retrotranscription was carried out by using the "ImProm-II Reverse Transcriptase Kit" (Promega). Briefly, annealing was performed at 25°C for 10 min and extension at 37°C for 1 h. Samples were inactivated at 70°C for 15 min and immediately subjected to real time PCR. Quantitative real time PCR was performed as previously described [8,14] in a Light Cycler apparatus (Roche) by using the "Light Cycler DNA-Master SYBR Green I Kit" (Roche). As PCR template, 2 μl of cDNA was used. Primer efficiency was verified by using serial dilution of cDNA ranging from 10^2 ^to 10^6 ^target copies per reaction (10^4 ^to 10^8 ^target copies per sample), and only oligonucleotides with comparable efficiency were chosen. Primers were designed to amplify segments of 100 to 150 bp and most were previously published [8,10,14]. The reference gene was *gyrB *and the reference condition was exponential phase of growth in TSB. Variation in gene expression was calculated by the 2^-ΔΔCT ^method [50] and statistical significance according to a more recent paper of the same authors [51].

## List of abbreviations

CSP: Competence stimulating peptide; *comC*: Competence stimulating peptide gene; ComD: Histidine kinase of the competence system; ComE: Response regulator of the competence system; BLP: Bacteriocin like locus peptide; BlpR: Response regulator of the bacteriocin like peptide locus; BlpH: Histidine kinase of the bacteriocin like peptide locus; w.t.: Wild type

## Authors' contributions

CT preformed experiments of microtiter biofilm model 1. LG set up microtiter biofilm model 2. DML performer the experiments of microtiter biofilm model 2. PJ performer experiments on continuous culture biofilm. CCK performer experiments on continuous culture biofilm. PE supervised the continuous culture biofilm and particpated in writing of the manuscript. FI supervised and performer construction of mutant. SR supervised model 2 and participated in writing of the manuscript. GP, participated in the study design. MRO supevised the work, defined the study design and carried out the writing of the manuscript. All authors read and approved the final manuscript.
